# Corrosion rate prediction and influencing factors evaluation of low-alloy steels in marine atmosphere using machine learning approach

**DOI:** 10.1080/14686996.2020.1746196

**Published:** 2020-06-19

**Authors:** Luchun Yan, Yupeng Diao, Zhaoyang Lang, Kewei Gao

**Affiliations:** a School of Materials Science and Engineering, University of Science and Technology Beijing, Beijing, China; b Beijing Advanced Innovation Center for Materials Genome Engineering, University of Science and Technology Beijing, Beijing, China

**Keywords:** Atmospheric corrosion, data mining, materials informatics, regression analysis, random forest, 106 Metallic materials, 212 Surface and interfaces, 404 Materials informatics / Genomics, corrosion

## Abstract

The empirical modeling methods are widely used in corrosion behavior analysis. But due to the limited regression ability of conventional algorithms, modeling objects are often limited to individual factors and specific environments. This study proposed a modeling method based on machine learning to simulate the marine atmospheric corrosion behavior of low-alloy steels. The correlations between material, environmental factors and corrosion rate were evaluated, and their influences on the corrosion behavior of steels were analyzed intuitively. By using the selected dominating factors as input variables, an optimized random forest model was established with a high prediction accuracy of corrosion rate (*R^2^* values, 0.94 and 0.73 to the training set and testing set) to different low-alloy steel samples in several typical marine atmospheric environments. The results demonstrated that machine learning was efficient in corrosion behavior analysis, which usually involves a regression analysis of multiple factors.

## Graphical Abstract

**Figure:**
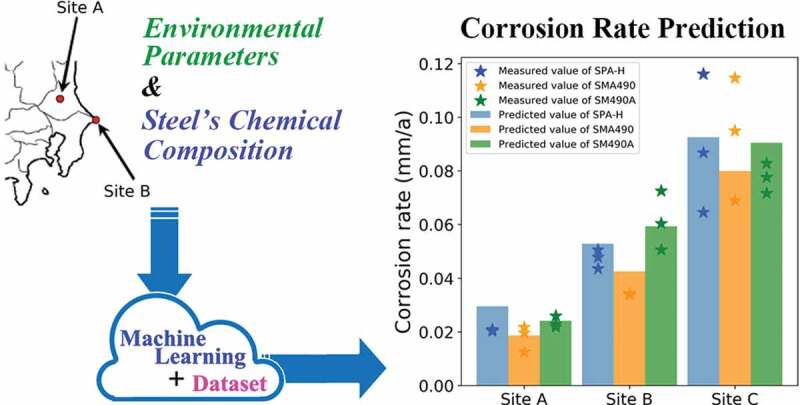

## 1. Introduction

Low-alloy steels are steels containing Cu, Cr, Ni, Mn, Si and P elements with a total concentration of 5 wt.% or less [1]. In recent years, low-alloy steels have been widely used as construction materials in marine environments due to their excellent physical properties and low cost. To understand the corrosion behavior of low-alloy steel in the marine atmosphere, factors that may affect the corrosion resistance have been extensively explored [2,3].

As an internal factor that directly determines the corrosion resistance of low-alloy steels, the functioning mechanisms of many alloying elements have been proven [4]. For instance, Diaz et al. [5] reported that the presence of high nickel (1–3 wt.%) contents in the steel raises the proportion of nanophase goethite in the inner rust layer, which increases its compactness and its corrosion resistance in moderate marine atmospheres. However, due to the effects of various environmental factors, the corrosion behavior of low-alloy steel in actual marine atmosphere environments is often very complicated [6]. Soares et al. [7] demonstrated that corrosion in marine atmosphere is primarily influenced by moisture and is accentuated by contaminants such as sodium chloride, and the corrosion rate was determined by the combined effects of different environmental factors. Considering the variety of low-alloy steels and the complexity of marine atmosphere environments, a systematic study of the material and environmental factors requires a large number of experiments [8]. But the atmospheric exposure test is a time-consuming and costly method [9]. Therefore, advanced experimental design methods and data analysis techniques are necessary.

The regression analysis can acquire a valid conclusion through only a limited number of experiments [10]. For instance, Panchenko et al. [11] proposed a forecast method of corrosion losses for a period of up to 50 years (with error within ± 30%) by regression analysis of 1-, 2-, 4- and 6-years’ corrosion data. In researches about the effects of exposure time, alloying elements, and environmental factors on corrosion resistance, the regression analysis is widely applied [12–14]. In conventional regression analysis, linear function, polynomial function and power function are mostly used [1,15]. Because of the limitations in dealing with multilevel structured data, conventional regression analysis is usually limited in case studies of particular steel’s corrosion behavior or the influence of individual factors [16]. Exploring the corrosion behavior in different environments is challenging because the corrosion process is influenced by multiple environmental factors simultaneously. For instance, Chico et al. [17] collected atmospheric corrosion data of 38 countries on four continents and made multiple linear regression analysis between corrosion rate and available environmental variables. Each environmental factor’s significance was only evaluated by its corresponding equation coefficient, and the *R^2^* between the equation predicted result and the actual measured result just reached 0.474. Therefore, corrosion data analysis urgently requires more advanced data mining methods.

In recent years, machine learning methods have attracted significant attention with their powerful data mining capabilities. Due to its enriched modeling packages and improved operability for non-professionals, it has also been abundantly used in material researches [18]. Some successful examples of the informatics-driven design of new materials include high-temperature alloys, low thermal hysteresis shape memory alloys, and metal additive manufacturing [19–21]. In the field of corrosion research, applications of machine learning methods such as support vector regression and artificial neural network were also reported [22,23]. These studies demonstrate the advantages of machine learning in correlation analysis, multivariate fitting, simulation, and data visualization [24–26]. Atmospheric corrosion data has typical characteristics such as multiple influencing factors, complex data distribution, and small data amount [17]. Compared with conventional regression analysis methods, machine learning methods have plentiful feature processing techniques, powerful regression ability, and robust generalization capacity to small data. Thus, machine learning provides better technical conditions for in-depth research and exploration of marine atmospheric corrosion [27].

In this study, a marine atmospheric corrosion database of low-alloy steels was utilized. Both statistical analysis and machine learning algorithms were employed to analyze the influence of alloying elements and environmental factors on the corrosion behavior of low-alloy steels. The application of machine learning methods in corrosion rate prediction was demonstrated, and its potential advantages were also suitably discussed.

## 2. Experimental details

### 2.1 Data and preprocessing

Corrosion Data Sheet (CoDS) from the National Institute of Materials Science (NIMS) MatNavi was used in this study [28]. This CoDS program on marine atmospheric corrosion comprises a group of alloy steels, which were exposed at three atmospheric exposure sites (Tsukuba, Choshi and Miyakojima in Japan) for 1, 2, 3, 5, 7 and 10 years, respectively. The information about materials (processing details, chemical compositions), corrosion properties (test conditions and specimens’ metal loss), environmental parameters and corresponding measuring methods were all recorded in detail. The dataset used in this study was composed of a part of the original CoDS: 306 rows of corrosion data with 18 alloy instances (Table 1), and each row was recorded in the form of 16 features (Table 2) and one target property (i.e. corrosion rate). Except for the 30 rows of corrosion data of steel Fe-9Ni and Fe-9Cr, the alloying elements content of the rest specimens were all below 5.2% (low-alloy steels). Due to the limited amount of corrosion data, we have retained these data. As a group of similar materials tested in the same environments, these data also may provide helpful information. The main purpose of this study was to investigate the feasibility of machine learning methods in solving corrosion problems. The effect of different alloying elements on the corrosion resistance of steels will be explored after collecting more related corrosion data. Therefore, only the total content of alloying elements was selected as a material feature. In the original CoDS, the 15 environmental features were recorded year by year. Because the exposure periods of specimens were different, the value of each environmental feature in Table 2 (TIME not included) denoted the average value of its corresponding measured values during the whole exposure period.

**Table 1.** Chemical compositions (wt.%) of selected low-alloy steels from CoDS of MatNavi.

Steels	%C	%Si	%Mn	%P	%S	%Cu	%Cr	%Ni
Fe-1Ni	0.001	<0.003	0.01	0.0003	0.0001	<0.01	<0.01	0.98
Fe-3Ni	0.001	<0.003	0.01	0.0005	0.0002	<0.01	<0.01	3.02
Fe-5Ni	0.001	<0.003	0.11	0.0006	0.0003	<0.01	<0.01	5.01
Fe-9Ni	0.001	<0.003	0.12	0.0005	0.0003	<0.01	<0.01	9.06
Fe-1Cr	0.005	<0.003	0.07	0.0010	0.0002	<0.01	1.01	<0.01
Fe-3 Cr	0.006	<0.003	0.05	0.0007	0.0001	<0.01	3.05	<0.01
Fe-5Cr	0.003	<0.003	0.11	0.0003	0.0010	<0.01	5.03	<0.01
Fe-9Cr	0.003	<0.003	0.12	0.0002	0.0003	<0.01	9.03	<0.01
Fe-0.5P	0.0012	<0.01	0.011	0.50	0.0004	0.009	<0.005	<0.003
Fe-1.0P	0.0022	<0.01	0.031	0.99	0.0005	0.015	<0.005	<0.003
Fe-1.5P	0.0024	<0.01	0.054	1.48	0.0004	0.021	<0.005	<0.003
Fe-0.4Cu	0.001	<0.01	<0.01	0.0006	0.0007	0.43	<0.005	<0.01
Fe-1Cu	0.0011	<0.01	<0.03	<0.001	<0.0003	1.00	<0.005	<0.003
Fe-2Cu	0.0011	<0.01	<0.03	<0.001	<0.0003	1.98	<0.005	<0.003
Fe-3Cu	0.0013	<0.01	<0.03	<0.001	<0.0003	2.97	<0.005	<0.003
SPA-H	0.089	0.22	0.39	0.10	0.0044	0.31	0.39	0.11
	0.09	0.43	0.38	0.102	0.005	0.30	0.67	0.18
SMA490	0.12	0.36	1.08	0.013	0.0076	0.34	0.51	0.08
	0.13	0.26	1.01	0.011	0.005	0.32	0.48	0.10
SM490A	0.15	0.285	1.45	0.020	0.0039	<0.01	0.05	<0.01
	0.14	0.25	1.35	0.012	0.003	<0.01	0.04	<0.01

For each of low-alloy steels SPA-H, SMA490 and SM490A, corresponding specimens may have two different chemical compositions as recorded in the CoDS database [28].

**Table 2.** List of considered material and environmental features.

Features		Data range	Descriptions
Material	ELEMENTS	0.5–9.2 wt.%	Total content of alloying elements
Environmental	T_MAX	31.0–37.0 °C	Maximum air temperature
	T_MIN	−8.0–9.5 °C	Minimum air temperature
	T_AVE	14.2–24.0 °C	Mean air temperature
	RH_MIN	15.0–55.0%	Minimum relative humidity
	RH_AVE	72.5–79.5%	Mean relative humidity
	SUNSHINE	1450 – 1990 h	Duration of sunshine
	TOW	3700 – 5300 h	Time of wetness
	PRECIPIT	1100 – 2300 mm	Precipitation
	WIND_MAX	5.5–39.5 m/s	Maximum velocity of wind
	WIND_AVE	1.1–4.7 m/s	Mean velocity of wind
	SOLAR	4100 – 6600 MJ/m^2^	Solar radiation
	UV	180 – 370 MJ/m^2^	Ultraviolet radiation
	CHLORIDE	2 – 55 mg NaCl/m^2^·d	Chloride deposition rate
	SO_2_	1.8–6.1 mg SO_2_/m^2^·d	SO_2_ deposition rate
	TIME	1, 2, 3, 5, 7, 10 years	Exposure period
Target property	Corrosion rate	0.0003–0.1995 mm/a	Annual corrosion depth, millimeter per year

Usually, due to differences in detection content and test conditions, data from different databases and literature cannot be directly combined into one dataset and used for model training. For example, in the original data record forms of CoDS database, the content of some trace alloying elements was not provided (or marked as below a detection threshold) for a few specimens, and the corrosion rate of a few samples was marked as larger than a certain value (due to the complete corrosion of specimen). To ensure the accuracy of machine learning, raw data will be pre-processed by filling-out missing values and correcting error data (i.e. data cleaning) [29]. In the chemical composition analysis of alloy steel, unmarked element composition usually means that it is absent or below a certain detection limit. In this study, we employed the smallest detection threshold value of the corresponding element in the whole dataset to fill the missing element content. Based on the domain knowledge, the exposure time-corrosion rate curve was fitted to complement the missing corrosion rate values for completely corroded specimens. Finally, all the material and environmental features were individually normalized within range 0 to 1.

### 2.2 Statistical and machine learning algorithms

#### 2.2.1 Pearson correlation coefficient

Pearson correlation coefficient is widely used to evaluate the degree of correlation between two variables, which is defined as the quotient of the covariance and their standard deviations, with values between −1 and 1. Normally, 1 represents a complete positive correlation, −1 represents a complete negative correlation, and 0 means completely irrelevant. For example, a Pearson correlation coefficient between 0.8 and 1.0 normally represents an extremely-strong correlation. However, the Pearson correlation coefficient could only capture the relation limited to linear function well [30].

#### 2.2.2 Maximal information coefficient (MIC)

In order to capture a wide range of associations both functional (e.g., linear function, exponential function, and periodic function) and not, MIC has been widely recognized and applied to correlation analysis [31,32]. For instance, Ahedo et al. [33] explore the possible relationships among the variables of the potentiodynamic anodic polarization test and the electrochemical potentiokinetic reactivation test using the MIC technique. MIC assigns a perfect score of 1 to all noiseless functional relationships (i.e. roughly equals the coefficient of determination, *R^2^* = 1.0), and a score of 0 to statistically independent variables.

#### 2.2.3 SHapley additive explanations (SHAP)

The SHAP method, which is based on a unification of ideas from the game theory and local explanations, proposes a rich visualization of individualized feature attributions that improves over classic attribution summaries [34]. Stojic et al. [35] successfully applied the SHAP feature attribution framework to examine the relevance of the monitored parameters and identify key factors that govern wet deposition of toluene, ethylbenzene and xylene. Each dot on the SHAP summary plot refers to a sample value, and the dots were colored by the value of that feature, from low (blue) to high (red). In order to get an overview of which features are most important for a model, SHAP values of each feature for every sample will be individually calculated. The positive SHAP value represents the ability to improve the target property, while a negative SHAP value represents the ability to reduce the target property. The importance of features was sorted by the sum of SHAP value magnitudes over all samples, and uses SHAP values to show the distribution of the impacts each feature has on the model output. More details can be obtained from the GitHub webpage of SHAP [36].

#### 2.2.4 Algorithms for corrosion rate prediction model

In this study, six statistical methods and machine learning algorithms were employed: Multiple Linear Regression (MLR), Ridge Regression (RR), Support Vector Regression (SVR), Random Forest (RF), Gradient Boosting Decision Tree (GBDT) and eXtreme Gradient Boosting (XGBoost). The MLR method is a classic statistical analysis method and it attempts to model the relationship between two or more explanatory variables and a response variable by fitting a linear equation to observed data. The RR method is a technique for analyzing linear regression and multiple regression data that suffer from multicollinearity. The SVR (which can efficiently perform a non-linear classification using what is called the kernel trick, implicitly mapping their inputs into high-dimensional feature spaces) and RF (which is an ensemble learning method that operates by constructing a multitude of decision trees) methods are two very well-known machine learning methods. The GBDT and XGBoost also belong to ensemble learning methods like the RF model, but they used different specific calculation strategies. In the literature, the mechanism of each method has been explained [29,37].

### 2.3 Experimental procedure

At first, correlation analysis (by combined analysis of the Pearson correlation coefficient and MIC results) was performed to evaluate the correlation between each feature and the corrosion rate. Only a part of these features would be selected as dominating features and was used for subsequent analysis and modeling. Then, RF algorithm was used to perform a regression analysis between the dominating features and the corrosion rate. Based on the RF results, the importance of each dominating feature to the corrosion rate was evaluated and discussed. Meanwhile, the SHAP method was also used to intuitively demonstrate the influence of these dominating features on low-alloy steel’s corrosion behavior. These experiments are mainly to verify the feasibility and advantages of machine learning in corrosion data mining.

On the other hand, the dataset was divided into two parts: the training set (80% amount) and the testing set (20% amount). The training set was mainly used for the optimization of model parameters, and the testing set was only used to verify the predictive performance of the optimized model. In the training step, the above mentioned six algorithms (i.e. MLR, RR, SVR, RF, GBDT and XGBoost) were individually applied to build a corrosion rate prediction model. The grid search scheme was used to determine the best model parameters, and the *k*-fold cross-validation (*k* = 5) was used (by dividing the training set into sub-training sets and validation set) to assess the predictive capabilities of each model with corresponding parameters [29,38]. Then, an optimized model with the best prediction capacity would be established. At last, the performance of the optimized corrosion rate prediction model was verified by the testing set.

All of the above statistical analysis and data mining work was conducted using Python software and scikit-learn toolkit.

### 2.4 Evaluation of model’s performance

Both coefficient of determination (*R^2^*, which directly evaluates two sets of data using a value between 0 and 1) and mean absolute error (*MAE*, which can reflect the error more intuitively) were employed for evaluation of the model’s prediction accuracy. They are formulated by the following equations:

(1) $$R^{2}=1-\frac{\sum_{i=1}^{n}{\left(f_{i}-y_{i}\right)}^{2}}{\sum_{i=1}^{n}{\left(y_{i}-\overset{ˉ}{y}\right)}^{2}}$$

(2) $$MAE=\frac{1}{n}\overset{n}{\underset{i=1}{\sum }}\left|f_{i}-y_{i}\right|$$

where *n* denotes the number of test samples, *f_i_* represents the predicted value, *y_i_* stands for the target value and $\overset{ˉ}{y}$ is the mean target value of all test samples.

## 3. Results and discussion

### 3.1 Selection of the dominating features

In order to reduce the complexity of the raw dataset and retain core information related to the corrosion rate, the features were screened before subsequent analysis and modeling. At first, features of multicollinearity are considered to provide redundant information regarding the target property (i.e. increase dataset’s complexity and reduce the result’s interpretability) [39]. Here, environmental features with significant correlation (by calculating Pearson correlation coefficient) were grouped into one cluster and they were considered to be multicollinearity features [29]. Meanwhile, the correlation between each feature and the corrosion rate was evaluated and ranked. For the features grouped into the same cluster, we selected only one feature having the strongest correlation with the corrosion rate as a representative feature of the corresponding cluster. Finally, the selected dominating factors will be both closely correlated with the corrosion rate and independent from each other.

**Figure 1.**
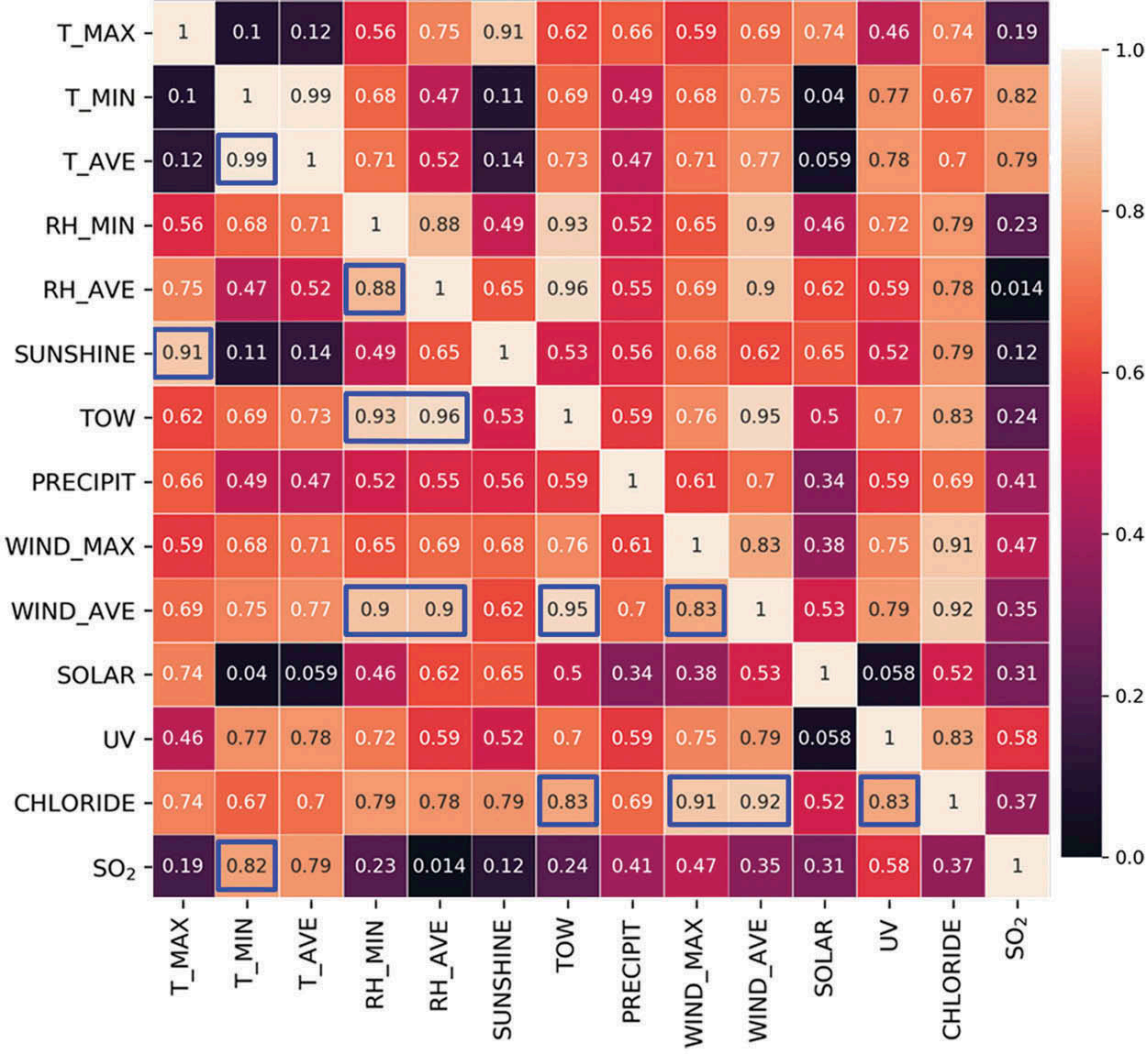
Pearson correlation map for environmental features. Pearson correlation coefficient is shown in each box, and the values indicating extremely strong correlation (>0.8) are marked with blue squares.

As depicted in Figure 1, the lighter the tone, the more significant is the corresponding correlation. Except for feature PRECIPIT and SOLAR having less correlation with others, four clusters of highly correlated features could be identified from the correlation map: T_MAX and SUNSHINE; T_MIN, T_AVE and SO_2_; RH_MIN, RH_AVE, TOW, WIND_MAX and WIND_AVE; WIND_MAX, WIND_AVE, UV and CHLORIDE. Here, features within the same group were considered multicollinearity features.

The possible relationship in each cluster could be inferred as that: a long term of sunshine exposure enhanced air temperature; deterioration of meteorological diffusion conditions and increase of heating exhaust emissions in winter cause the increase of atmospheric sulfur dioxide content; long term of high relative humidity normally increased the time of wetness, and strong wind-induced splashes increase relative humidity in the air; wind promoted the splashing and diffusion of salt ions in the ocean [40], and strong ultraviolet radiation (i.e. strong sunlight exposure) will accelerate the evaporation of seawater (i.e. the transfer of chloride ions to the atmosphere). The above reasons are our simple speculations, and actual mechanisms require further professional in-depth investigations.

**Figure 2.**
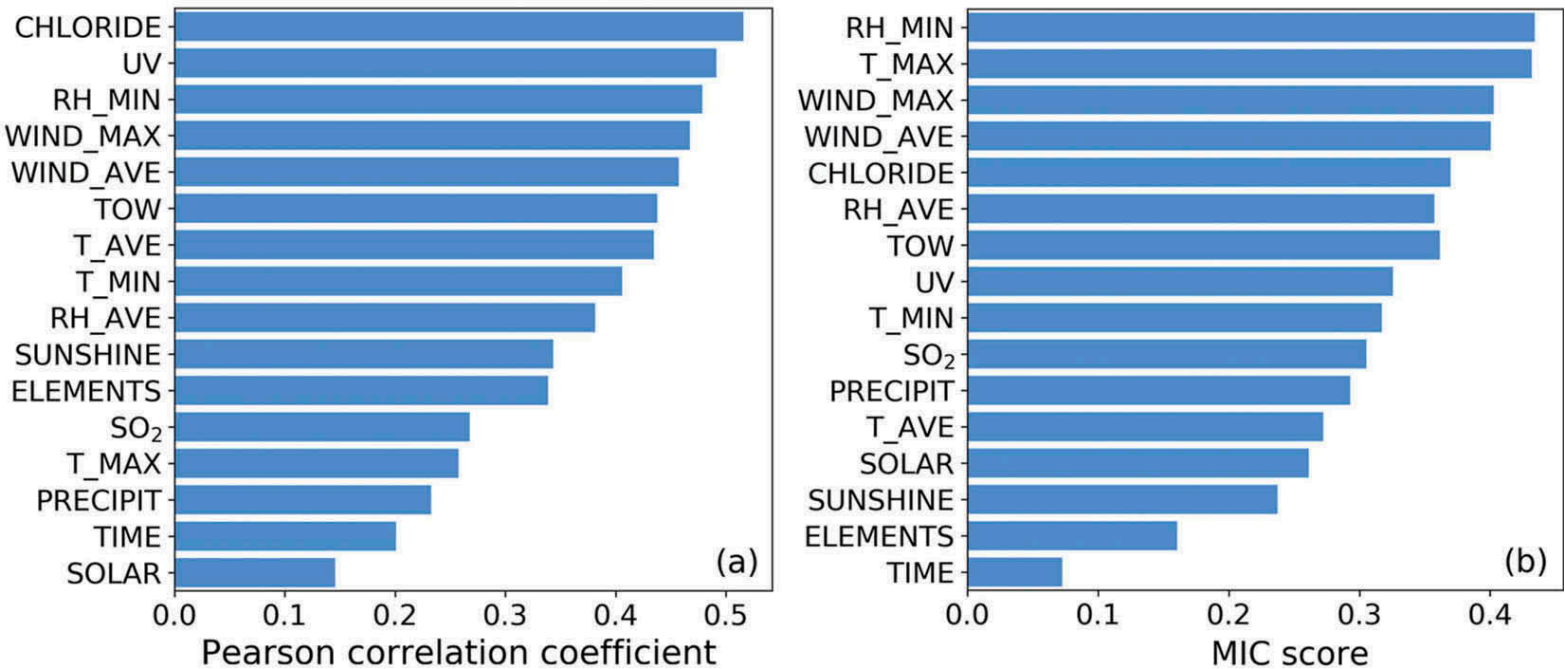
Correlation coefficient between each feature and the corrosion rate from (a) Pearson correlation coefficient method and (b) maximal information coefficient (MIC) method.

As shown in Figure 2, each feature’s significance to the corrosion rate was ranked. Since it was uncertain whether the input feature was linearly (or non-linearly) related to the corrosion rate, both the Pearson correlation coefficient method and the MIC method were employed. To reduce dataset complexity, only one feature, which had the strongest correlation with the corrosion rate, would be selected from each of the above four clusters of multicollinearity features. Thus, features T_MAX, T_AVE, RH_MIN and CHLORIDE were selected from each cluster. Since SO_2_ becomes the common air pollutant in some coastal areas and researches has proved its distinct effect on the corrosion rate of atmospheric corrosion, the feature SO_2_ was also selected [41]. Including the above-mentioned features without multicollinearity (i.e. ELEMENTS, TIME, PRECIPIT and SOLAR), a total of nine features (ELEMENTS, T_MAX, T_AVE, RH_MIN, PRECIPIT, SOLAR, CHLORIDE, SO_2_ and TIME) were finally selected as the dominating factors of the corrosion rate. In the subsequent analysis and modeling, only the influences of these nine features were explored.

### 3.2 Corrosion behavior analysis with random forest algorithm

In most cases, machine learning model works like a black box. It always lacks a theoretical explanation of the relationship between the input and output variables [42]. Fortunately, some machine learning algorithms are trying to enhance this functionality. Unlike the Pearson correlation coefficient and MIC, which only emphasize the degree of correlation rather than the degree of numerical change, the RF method can directly evaluate the feature importance by measuring the value change of the target property due to the changes in a feature value [43].

**Figure 3.**
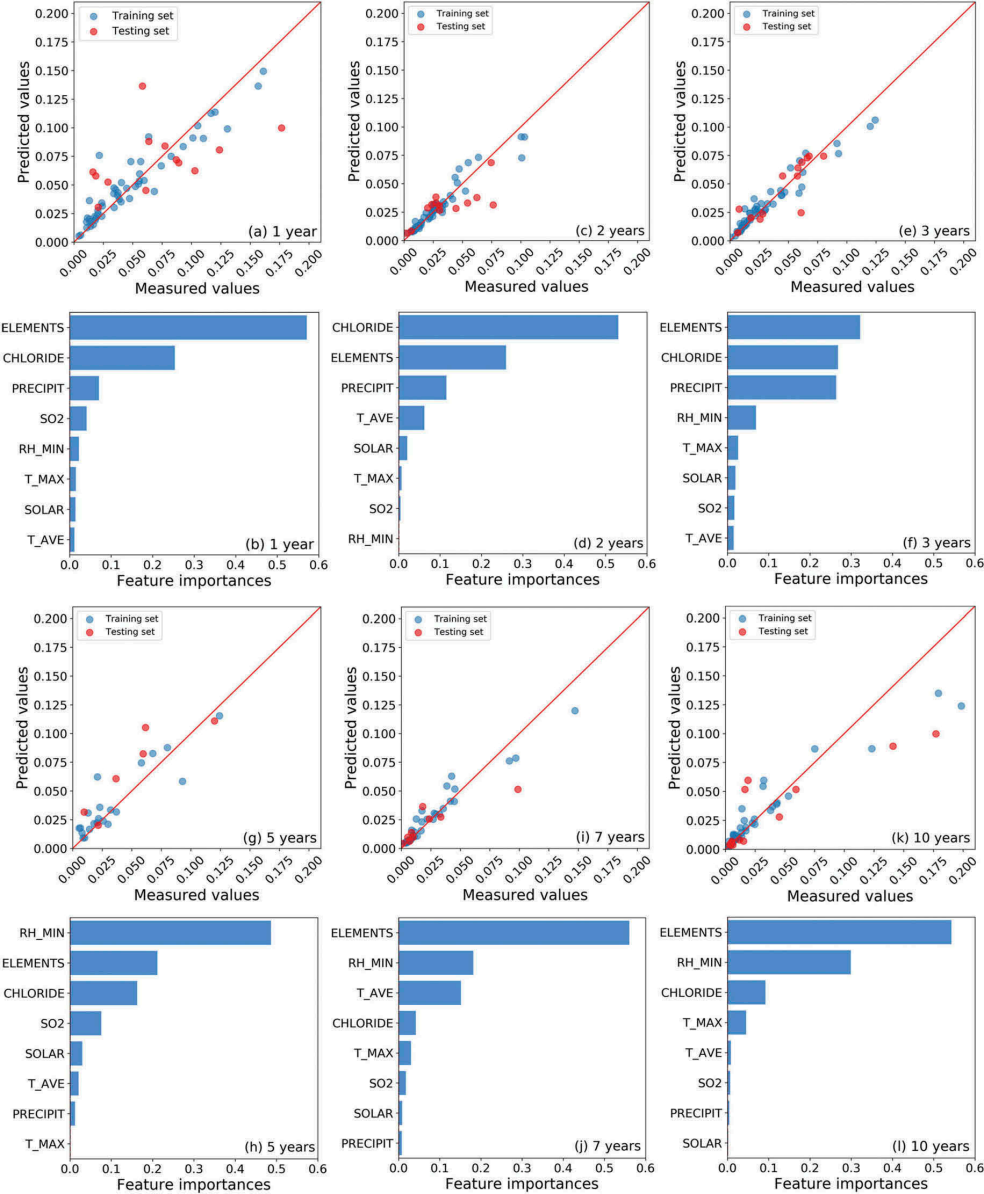
The predictive accuracy (predicted corrosion rates *vs*. measured corrosion rates) of the random forest model and the feature importance of corresponding input variables. The random forest models were separately built for samples with (a-b) 1, (c-d) 2, (e-f) 3, (g-h) 5, (i-j) 7 and (k-l) 10 years of exposure.

As depicted in Figure 3, a RF model was trained and optimized for samples of different exposure periods. Since the rust layer has a very complicated effect on the steels, the original dataset was divided into six groups by their exposure periods (only in this section) [44,45]. Through a comparison of the predicted corrosion rates and measured values, the RF models for all the exposure periods exhibited good predictive accuracy. Since there was no obvious over-fitting (e.g., a significant difference in the prediction accuracy of the training set and testing set), we assumed that the model successfully fitted the relationship between input features and target property. Then, the feature importance derived from the model was considered to be reliable.

During the whole exposure periods (Figure 3), the total content of alloying elements (ELEMENTS) was always one of the most significant features. Meanwhile, as shown in Figure 3b,d and f, the chloride deposition rate (CHLORIDE) and precipitation (PRECIPIT) had the most significant effect on the corrosion rate in the first three years of exposure test. In the initial formation stage of the rust layer, the corrosion product film was loose and thin. The deposited chloride increased the concentration of corrosive ions, and the precipitation (in the forms of drizzle, rain, sleet, snow, graupel and hail) easily penetrated through the rust layer and created a wet corrosive environment on the metal surface. In more than five years of exposure tests, the RH_MIN became the most significant environmental factor (Figure 3(h,j,l)). As a thick and dense rust layer had been formed on the surface of the specimen, its permeability had changed significantly [46]. It became difficult for both the chloride ions and raindrops to reach the metal surface by permeating the rust layer. However, the long-term high relative humidity would affect the water content in the rust layer and help to form a corrosive microenvironment on the metal surface [45]. For instance, Ma et al. [47] proved that the existence of the outer layer makes the time of wet longer in the rust/steel interface, which provides a suitable location for electrochemical reactions, thereby inducing incessant corrosion and poor weatherability.

### 3.3 Data mining with SHAP method

**Figure 4.**
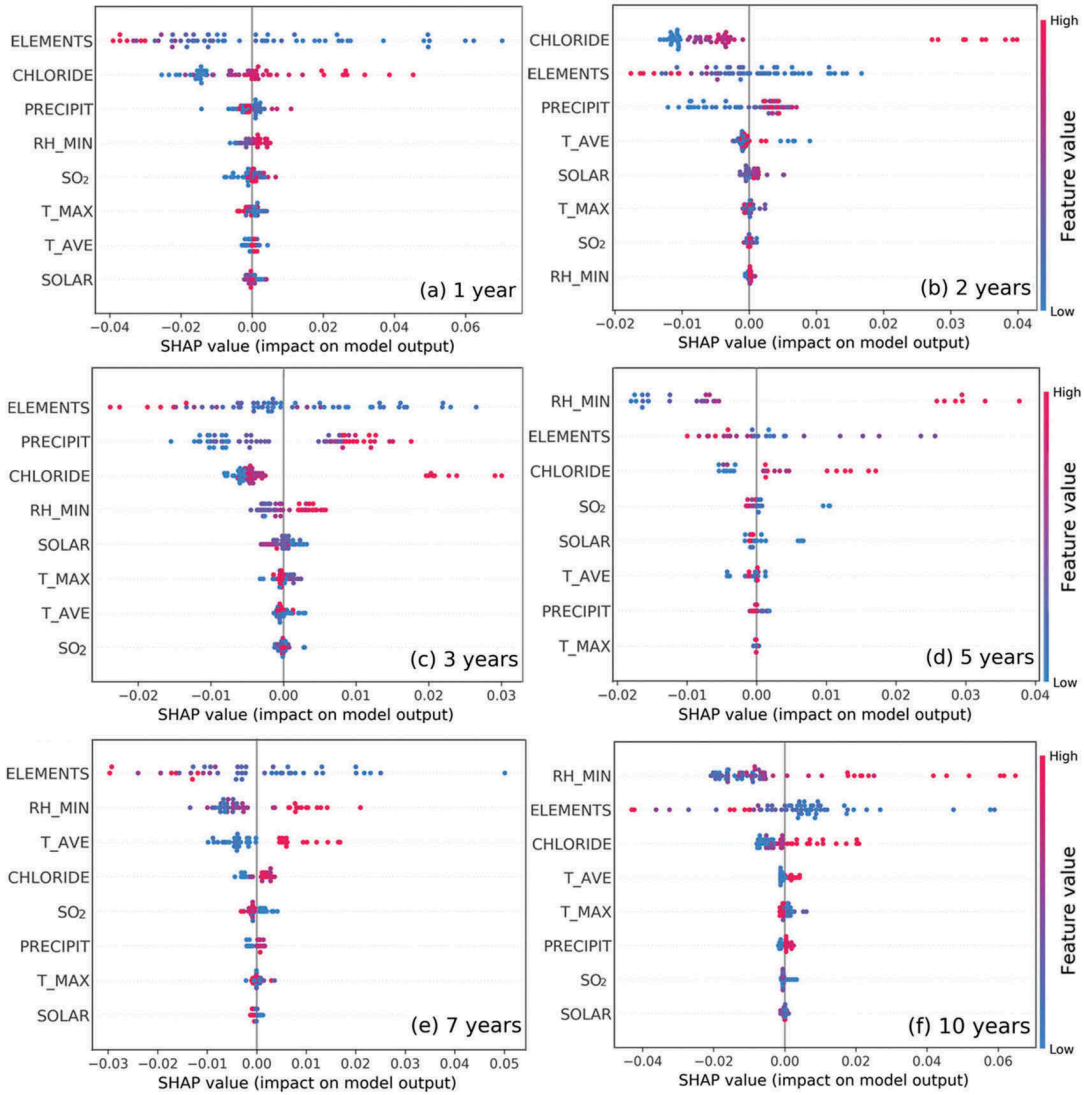
The SHAP variable importance plot of marine atmospheric corrosion data in different exposure periods. Each dot refers to a sample, and the dots were colored by the corresponding feature value from low (blue) to high (red). The positive SHAP value represents the ability to increase the corrosion rate, and the negative SHAP value represents the ability to decrease the corrosion rate. The features were listed on the vertical axis from top to bottom in a sequence of their feature importance.

In order to further analyze the functioning mechanism of each feature, this study also used a SHAP algorithm for data mining. As shown in Figure 4, the features were arranged on the vertical axis based on their importance from high to low. The feature importance results of each exposure period were almost the same with the results of the above RF model. Each dot on the plot referred to a specimen, and these dots were colored from blue to red according to its corresponding feature value (from low to high). For example (Figure 4(a)), the SHAP value changed from positive to negative when the value of feature ELEMENTS changed from small to large (i.e. from blue to red). As the more positive SHAP value represents the higher the corrosion rate (and the more negative SHAP value represents the lower the corrosion rate), it indicated that an increase in the total alloying elements content improves the corrosion resistance.

Through the same way of analysis, it could be found that the increase of CHLORIDE, PRECIPIT, RH_MIN distinctly accelerated the corrosion rate. This conclusion was consistent with our domain knowledge. Compared with these four significant factors, the other features had a weak effect on the corrosion rate. The SHAP method displayed the functioning mechanism of these features in a very intuitive way. Especially when the working mechanism of the target feature was not clear, the SHAP method would be a quite effective tool.

### 3.4 Modeling and application of corrosion rate prediction models

By using the nine selected factors in section 3.1 as input variables and the corrosion rate as output variable, six statistical and machine learning algorithms were used to establish the corrosion rate prediction models. For each model, the optimized parameters were: MLR, corrosion rate = 0.1357 T_MAX – 0.1836 T_AVE + 0.3566 RH_MIN – 0.1420 PRECIPIT + 0.0142 SOLAR + 0.3101 CHLORIDE – 0.1547 SO_2_ + 0.0010 TIME −0.2406 ELEMENTS + 0.0102; RR, alpha = 0.85; SVR, kernel = rbf, C = 100, gamma = 0.05; RF, n_estimators = 10; GBDT, alpha = 0.9, learning_rate = 0.1, n_estimators = 100; XGBoost, booster = gbtree, learning_rate = 0.1, n_estimators = 100.

**Figure 5.**
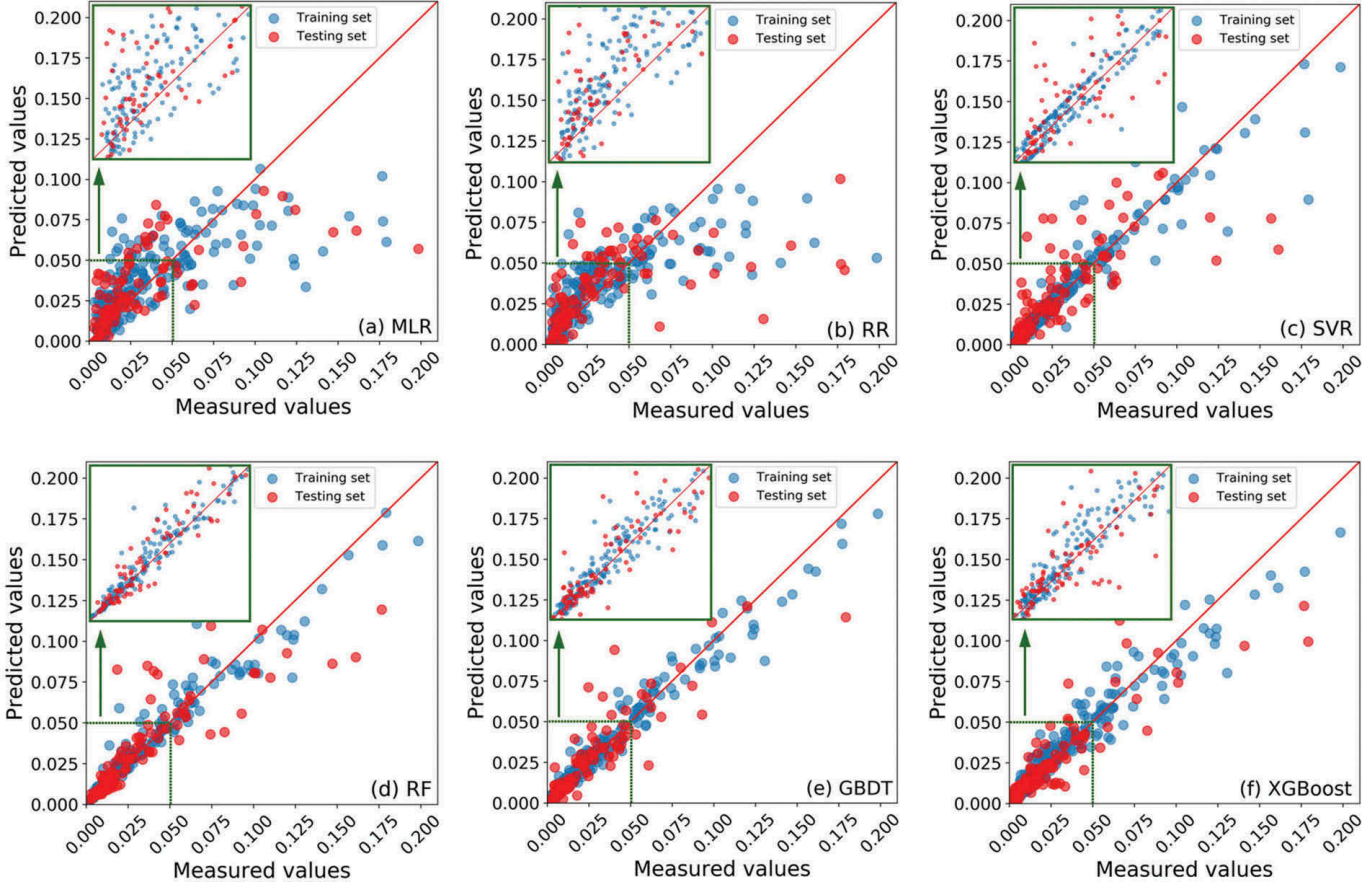
The performance of the machine learning models using (a) Multiple Linear Regression (MLR), (b) Ridge Regression (RR), (c) Support Vector Regression (SVR), (d) Random Forest (RF), (e) Gradient Boosting Decision Tree (GBDT) and (f) eXtreme Gradient Boosting (XGBoost) algorithms.

Figure 5 presents the corrosion rate prediction results of the optimized models. The predicted corrosion rate is plotted as a function of the measured corrosion rate. For a perfect model, the predicted corrosion rate will be exactly the same as the measured corrosion rate and all the data points will fall along with the 45° diagonal line in the plot. For regression tasks with many input features, statistical analysis methods such as MLR and RR had a poor fitting effect. The SVR model was much improved, but a distinct accuracy difference between the training set and testing set was observed. So, it probably was over-fitted. Meanwhile, the RF, GBDT and XGBoost models showed better predictive accuracy. As listed in Table 3, the *R^2^* and *MAE* results of each optimized model to both the training set and the testing set were also calculated. The well generalization ability of machine learning algorithms makes it having better prediction ability than traditional statistical methods. But the training set in this study covered a group of material and environmental factors, and we think that its data amount was insufficient to fully reflect the impact of these factors on corrosion rate. Therefore, the model’s prediction accuracy to the testing set (i.e. totally new combination of material and environmental factors) was reasonably lower than the training set. The RF, GBDT and XGBoost models all showed close prediction accuracies. As a very typical algorithm, we used the RF model in the subsequent test.

**Table 3.** The predictive accuracy of machine learning models. The coefficient of determination (*R^2^*) and mean absolute error (*MAE*) were individually calculated for samples in the training set and testing set.

	*R^2^*		*MAE* (mm/a)	
Models	Training set	Testing set	Training set	Testing set
Multiple Linear Regression	0.54	0.43	0.015	0.018
Ridge Regression	0.56	0.33	0.014	0.019
Support Vector Regression	0.90	0.48	0.006	0.014
Random Forest	0.94	0.73	0.004	0.010
Gradient Boosting Decision Tree	0.96	0.69	0.005	0.009
eXtreme Gradient Boosting	0.93	0.77	0.006	0.009

It could be found that every model had better prediction accuracy for samples with a low corrosion rate (subplots in Figure 5). For example, the prediction accuracy of the RF model significantly decreased when the measured corrosion rate was higher than 0.050 mm/a (a = annum = year). A significant difference in sample quantity also could be observed from the sub-plots. Since the main functioning mechanism of the machine learning model was to extract the underlying information from the dataset, the data quantity would seriously influence the model’s regression effect. Therefore, it is necessary to add more relevant data to improve the corresponding prediction accuracy.

**Figure 6.**
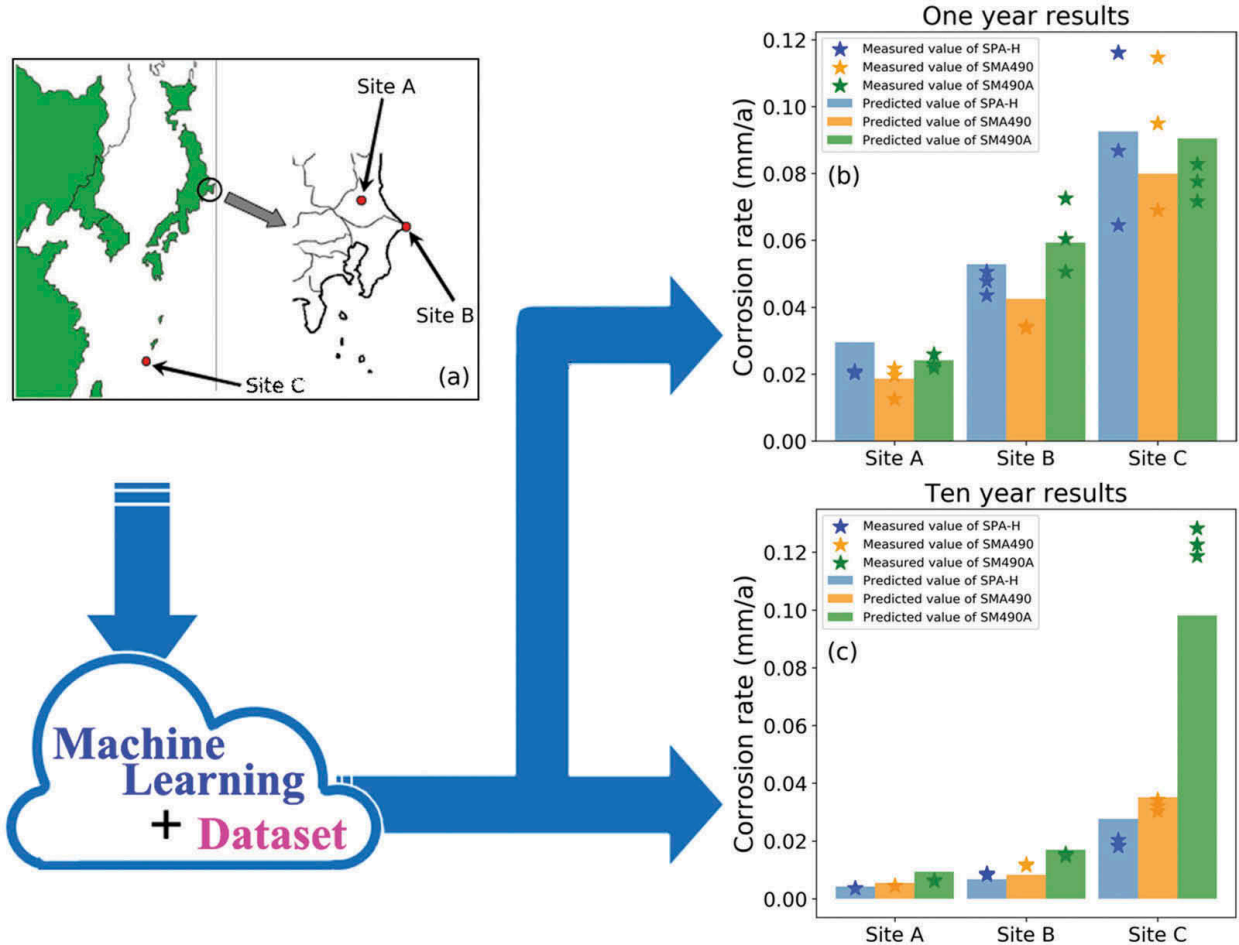
The application of machine learning models for corrosion resistance evaluation on the basis of very limited material and environmental information. (a) illustration of three different atmospheric exposure sites [28]; the measured corrosion rate (star icons, three parallel samples) *vs*. predicted corrosion rate (bar plot) of typical low-alloy steel SPA-H, SMA490 and SM490A after (b) one and (c) ten years of exposure.

Although there is a large amount of corrosion data in the relevant databases and literature, their testing environments are usually different. The errors introduced by the difference in testing environments will be further amplified after the regression analysis. When exploring the effect of some specific factors (e.g., alloying elements) on the steel, the obtained results are probably unreliable [1,2,17]. Therefore, we proposed a simulation method for the conversion and utilization of corrosion data from a wide range of sources. As depicted in Figure 6(a), there were three atmospheric exposure sites in different geographic locations. After collecting the corresponding environmental parameters and the chemical composition of the desired test material (e.g., low-alloy steels SPA-H, SMA490 and SM490A), they would be transferred to the optimized RF model as input data. Then, the possible corrosion rate of these steels in each location would be immediately obtained. Since we used the exposure period as one of the input features, the results in a certain exposure period also could be directly calculated (bar plots in Figure 6(b–c)). Compared with the actual measured data (star icons, three parallel samples of each steel, a group of new data from the CoDS), this strategy was demonstrated to be feasible. At the current stage, it was only an idea because of the limitations on the data amount. It was believed that the accuracy and robustness of this method could be further improved by enriching the database (i.e. covering more steel types and marine atmospheric environments in different geographical regions).

### 3.5 Discussions on machine learning in corrosion data analysis

The main purpose of this study is to explore the technical characteristics and potential applications of machine learning in the field of corrosion research. Firstly, the results of multicollinearity and correlation evaluation (e.g., the Pearson correlation coefficient and MIC) helped identify the most relevant features to the corrosion rate. Then, the RF algorithm quantified the importance sequence of the dominating features and the SHAP method intuitively displayed their influence on the corrosion behavior. Compared with conventional analytical methods, machine learning provided more information in various forms. On the other hand, the application of machine learning in corrosion rate prediction was demonstrated. The machine learning algorithm showed better regression ability for data with multiple variables, such as the marine atmospheric corrosion data. Based on the performance of this corrosion rate prediction model, it is considered as an effective tool for further corrosion research, such as service life estimation, corrosion resistance evaluation and alloy composition optimization.

As a popular big data processing method, machine learning has significant advantages in data mining. But because the amount of corrosion data is usually small, the advantages and necessity of machine learning are not always significant. Usually, the data quantity and quality are considered the accuracy guarantee of machine learning analysis and modeling [48]. But due to differences in corrosion test standards and environmental monitoring methods, atmospheric corrosion data in the literature are difficult to summarize and use directly [17,49]. It is still challenging to collect enough atmospheric corrosion data, which can adequately represent the steel and environment characteristics. For the unclear phenomena and undiscovered laws, machine learning only provides some exploratory analysis results. The real mechanism still needs to be verified and analyzed through professional experiments and guidance of domain knowledge.

## 4. Conclusions

Based on the atmospheric corrosion data collected from the NIMS database, the correlation between material, environmental factors and corrosion rate was explored by using both the Pearson correlation coefficient and maximal information coefficient. Nine factors including alloying elements content, maximum air temperature, minimum air temperature, minimum relative humidity, precipitation, solar radiation, chloride deposition rate, SO_2_ deposition rate and exposure period were identified as dominating factors in determining the corrosion rate. Then by using the random forest algorithm and the SHapley Additive exPlanations algorithm, the importance sequence of the nine factors to corrosion rate was analyzed. We also intuitively demonstrated that the corrosion rate in the marine atmosphere was primarily influenced by chemical compositions, chloride deposition rate and precipitation in the first three years of exposure test. Then probably due to the formation of a thick and stable rust layer, the relative humidity became the most significant environmental factor. Meanwhile, by comparing different machine learning algorithms, a random forest algorithm-based corrosion rate prediction model was established. The optimized model was proved to have high prediction accuracy for multiple steel samples in different environments. The advanced regression ability and data mining capacity of machine learning were proven to be very useful in corrosion data analysis.

## Data Availability

The raw data required to reproduce these findings are available to download from https://smds.nims.go.jp/corrosion/index_en.html (Homepage of CoDS, NIMS).
